# Influences of *De Qi* induced by acupuncture on immediate and accumulated analgesic effects in patients with knee osteoarthritis: study protocol for a randomized controlled trial

**DOI:** 10.1186/s13063-017-1975-7

**Published:** 2017-06-05

**Authors:** Min Li, Hongwen Yuan, Pei Wang, Siyuan Xin, Jie Hao, Miaomiao Liu, Jinfeng Li, Man Yu, Xinrui Zhang

**Affiliations:** 1 Department of Acupuncture and Physical Therapy, Beijing Luhe Hospital Affiliated to Capital Medical University, No.82, Xinhua south street, Tongzhou District, Beijing, 101149 China; 2 School of Traditional Chinese Medicine, Capital Medical University, No.10, Xitoutiao, Outside of Youanmen, Fengtai District, Beijing, 100069 China; 3 Teaching and Research Section of Acupuncture-moxibustion and Tuina, Chengde Medical College, Shangerdaohezi, Shuangqiao District, Chengde, Hebei Province 067000 China; 4 National Institute of Complementary Medicine, Western Sydney University, Locked bag 1797, Penrith, 2751 Sydney Australia

**Keywords:** *De Qi*, *Qi* arrival, knee osteoarthritis (KOA), visual analog scale (VAS), randomized controlled trial

## Abstract

**Background:**

*De Qi* is a special sensational response upon acupuncture needling. According to traditional acupuncture theory, the treatment is “effective only after *Qi* arrival”; that is, *De Qi* is an important indicator of therapeutic efficacy and good prognosis. However, it is still disputable whether *De Qi* improves the efficacy of acupuncture therapy. This prospective, randomized controlled trial aims to explore the influence of *De Qi* induced by acupuncture on immediate and accumulated analgesic effects in patients with knee osteoarthritis (KOA).

**Methods/design:**

Eighty-eight patients with KOA will be recruited and randomly assigned to the *De Qi* group (enhanced stimulation to evoke *De Qi*) and the control group (weak stimulation to avoid *De Qi*) in the Department of Acupuncture and Physical Therapy, Beijing Luhe Hospital Affiliated to Capital Medical University. Each patient will receive three 30-minute sessions per week for 4 consecutive weeks and undergo a 1 month follow-up. The severity of knee pain, as measured on a 100-mm visual analog scale (where 0 indicates no pain and 100 indicates intolerable pain) will be used as the primary outcome, and the Knee injury and Osteoarthritis Outcome Score will be used as the secondary outcome. Both indexes will be measured before and after the 1^st^ (for evaluating the immediate analgesic effects), 3^rd^, 6^th^, 9^th^, and 12^th^ (for evaluating the accumulated analgesic effects) treatments and at the end of the follow-up. The intensity of the *De Qi* sensation will be assessed by the Chinese-Modified Massachusetts General Hospital Acupuncture Sensation Scale at the end of each treatment. Side effects during the treatments will be recorded and analyzed as well. The comparisons between the *De Qi* group and the control group will be done by using both an intention-to-treat analysis and a per-protocol analysis.

**Discussion:**

This prospective randomized controlled study will be helpful in enhancing our understanding of the analgesic effect of *De Qi* on patients with KOA and may provide a clinical basis for further investigation of the relationship between *De Qi* and the therapeutic efficacy of acupuncture, thereby offering some evidence for the role of *De Qi* in an efficacious acupuncture therapy.

**Trial registration:**

Chinese Clinical Trial Registry, ChiCTR-IIR-16008972. Registered on 4 August 2016 Additional file 2.

**Electronic supplementary material:**

The online version of this article (doi:10.1186/s13063-017-1975-7) contains supplementary material, which is available to authorized users.

## Background

As one key component of traditional Chinese medicine, acupuncture has made indelible contributions to human health during the thousands of years’ development of medicine. *De Qi*, also called “*Qi* Arrival” in ancient literature and recently “acupuncture sensation,” refers to the reaction of meridian *Qi* at the needling site after the insertion of the needle to a certain depth with various manipulations by the acupuncturist, namely, lifting, thrusting, twisting, and rotating. At the same time, the patients often perceive soreness, numbness, fullness, and heaviness, while the acupuncturists often experience a sensation of heaviness, tightness, or stagnation at the hand [1].

Considered as an important indicator of therapeutic efficacy, *De Qi* has been emphasized by acupuncture practitioners over the ages. In the meanwhile, researchers have attempted to investigate the correlation between *De Qi* and the therapeutic efficacy of acupuncture. While many studies have suggested that *De Qi* helped improve the efficacy [2–4], some high-quality studies concluded otherwise [5–7]. As of today, it remains controversial whether or not *De Qi* contributes to the therapeutic effect of acupuncture [8–10]. In light of recent developments of evidence-based medical research and the related investigative techniques, this topic has become a focus of attention in the modern acupuncture field [11, 12].

Knee osteoarthritis (KOA), also called degenerative arthritis and osteoarthritis, is a chronic joint disorder caused by degenerative disease in the knee synovium. Characterized by degeneration of articular cartilage and osteosclerosis/hyperosteogeny [13], it is the most common chronic, progressive, and degenerative joint disease in the population aged 40 and older. The main clinical manifestations of KOA include aching, movement disorders, and joint deformation [14]. It has become a growing concern for public health in aging societies worldwide. According to an epidemiological study from Manchester University, UK, KOA is the 4^th^ and the 8^th^ cause of labor loss in males and females, respectively, in western countries. Epidemiological data from China showed that KOA occurred in 85% of people more than 65 years old [15]. So far there is no especially effective treatment for this disease. From the point of view of traditional Chinese medicine, KOA can be categorized as a bone impediment. According to multiple research studies, acupuncture has a good therapeutic efficacy on bone impediments, including KOA [16–18] Additional file 2.

In this research, we will use both strong stimulation to evoke *De Qi* and weak stimulation to avoid *De Qi* during the acupuncture treatment of KOA. We will compare the immediate and the accumulated analgesic effects between the two groups in order to explore the correlation between *De Qi* and the therapeutic efficacy of needling at certain acupoints, thereby providing a clinical basis for further mechanistic studies of *De Qi* Additional file 2.

## Methods/design

### Overview

**Fig. 1:**
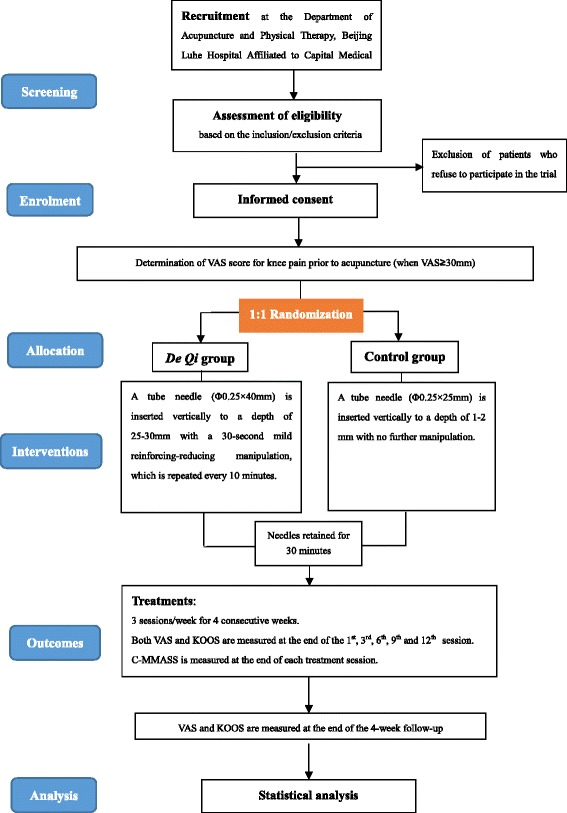
Flow chart of the trial. *KOA* knee osteoarthritis, *VAS* visual analog scale, *KOOS* Knee injury and Osteoarthritis Outcome Score, *C-MMASS* Chinese-Modified Massachusetts General Hospital Acupuncture Sensation Scale

This study is a prospective, randomized controlled trial that compares the therapeutic outcomes in the presence and absence of *De Qi*. The trial will be carried out at the Department of Acupuncture, Beijing Luhe Hospital Affiliated to Capital Medical University, Beijing, China Additional file 2. The procedure will last 14 weeks, which consists of a period of pre-randomization adaptation for 2 weeks, followed by a 4-week acupuncture therapy after the randomization, and finally a 1 month follow-up (Fig. 1) Additional file 2.

The trial follows strictly the guidelines of the Declaration of Helsinki (Version 2000) and has been approved by the Ethics Committee of Beijing Luhe Hospital Affiliated to Capital Medical University (Approval Number 2016-LH-WZ-002). Each patient will be informed both orally and in writing with complete details about the procedure, the possible risks, the voluntary nature of the participation, and the right to withdraw at any moment prior to enrollment Additional file 2. Informed consent will be required to be signed by each patient before his/her entry into the trial and will be kept in the research archives.

### Subjects

A total of 88 patients diagnosed with KOA will be included in the trial. The diagnostic criteria for KOA are as defined by the American College of Rheumatism [19]: (1) knee pain most of the time; (2) crepitus in motion; (3) less than 30 minutes of morning stiffness; (4) patient more than 38 years old; (5) bone enlargement. Patients who meet criteria (1)–(4) or (1) + (2) + (5) or (1) + (4) + (5) are diagnosed with KOA.

### Inclusion criteria

Patients to be recruited in this study should meet the diagnostic criteria, which include: diagnostic criteria of KOA; age 30–70 years; recurrent pain in unilateral or bilateral knee joint(s) for more than 1 month; average intensity of knee pain measured by a 0–100 mm visual analog scale (VAS ) equal to or greater than 30 mm in the last month before randomization; good health in general; not receiving medication or other treatments for at least 2 weeks prior to inclusion into the trial; written consent to participate in the trial Additional file 2.

### Exclusion criteria

Patients with any of the following conditions will be excluded: manifestation of joint space narrowing, chondral sclerosis and/or cystic degeneration, osteophyte formation at the edge of the joint; infectious, gouty, rheumatoid, or traumatic arthritis; known life-threatening diseases, mental illnesses, or neurological disorders such as Alzheimer’s disease and Parkinson’s disease; pregnancy or anticipation of pregnancy; concurrence of pain(s) on other site(s); inability to complete the treatment course or to be evaluated for therapeutic efficacy Additional file 2.

### Sample size

Patients with KOA will be randomized into two groups in a 1:1 ratio. The sample size for each group was calculated according to the following equation for a design with repeated measures [20] Additional file 2:

$$ \mathrm{n}=\frac{2\kern0.5em *\kern0.5em {\upsigma}^2\kern0.5em {\left({\mathrm{u}}_{\alpha}\kern0.5em +\kern0.5em {\mathrm{u}}_{\upbeta}\right)}^2}{\updelta^2}\left\{\left[\frac{1}{\mathrm{r}}+\left(1-\frac{1}{\mathrm{r}}\right)\uprho \right]-\frac{\uprho^2}{1/ P+\left(1-1/ P\right)\rho}\right\} $$

where *P* stands for the number of observations prior to acupuncture (VAS score before the first intervention, one time point before treatment), and *r* is the observation number after acupuncture (VAS scores at the end of the 1^st^, 3^rd^, 6^th^, 9^th^, and 12^th^ treatments, a total of five time points after treatment). The values of ρ, α, μ_α_, and μ_β_ used here are as follows: ρ = 0.70 (usually between 0.50 and 0.75); α = 0.05 (bilateral); μ_α_ = 1.96; μ_β_ = 0.8417. As a result, an estimated sample size of 37 patients per group was obtained, assuming that a minimal difference of δ = 10 mm is considered as a clinically significant relief of chronic pain, as recommended by IMMPACT [21], and with a standard deviation (σ) of 29.5 mm of the VAS score for knee pain in all patients prior to treatment, according to the data from the clinical trial run by White et al. [22]. Considering a possible loss to follow-up of 20% Additional file 2, we planned to include total of 88 patients in our study.

### Randomization and blinding

A table of random numbers will be generated by a third party who will not participate in the trial, using SPSS 20.0 software (SPSS Inc., Chicago, IL, USA) Additional file 2. The randomization program will be concealed in an opaque envelope and managed by the third party. At 5–10 minutes prior to the acupuncture treatment, the acupuncturist will inform the manager of the randomization program about the patient number and name via telephone; the acupuncturist will then be informed of the patient’s randomization number and treatment group. Only the acupuncturist is permitted to contact the manager Additional file 2.

A three-way separation (patients, the acupuncturist, and the recorder) will be performed for blinding. All patients as well as the recorder will be blinded to information about the randomization and the received treatment, which is only known to the acupuncturist. During the treatment, the recorder will be isolated from the operator with a screen and ear plugs. In addition, the *De Qi* group and the control group will be represented as A and B, respectively, for data analysis to ensure the objectivity of the statistician from the third party who does not participate in the procedure. Before analysis, all data will be kept by designated personnel. Unblinding will not be done until the completion of data analysis Additional file 2.

### Researchers

Treatments will be performed by a licensed acupuncturist with more than 6 years’ experience. Two supervisors will monitor the procedure throughout the study. The acupuncturist and other researchers responsible for data collection will be trained before the trial.

### Interventions

**Fig. 2:**
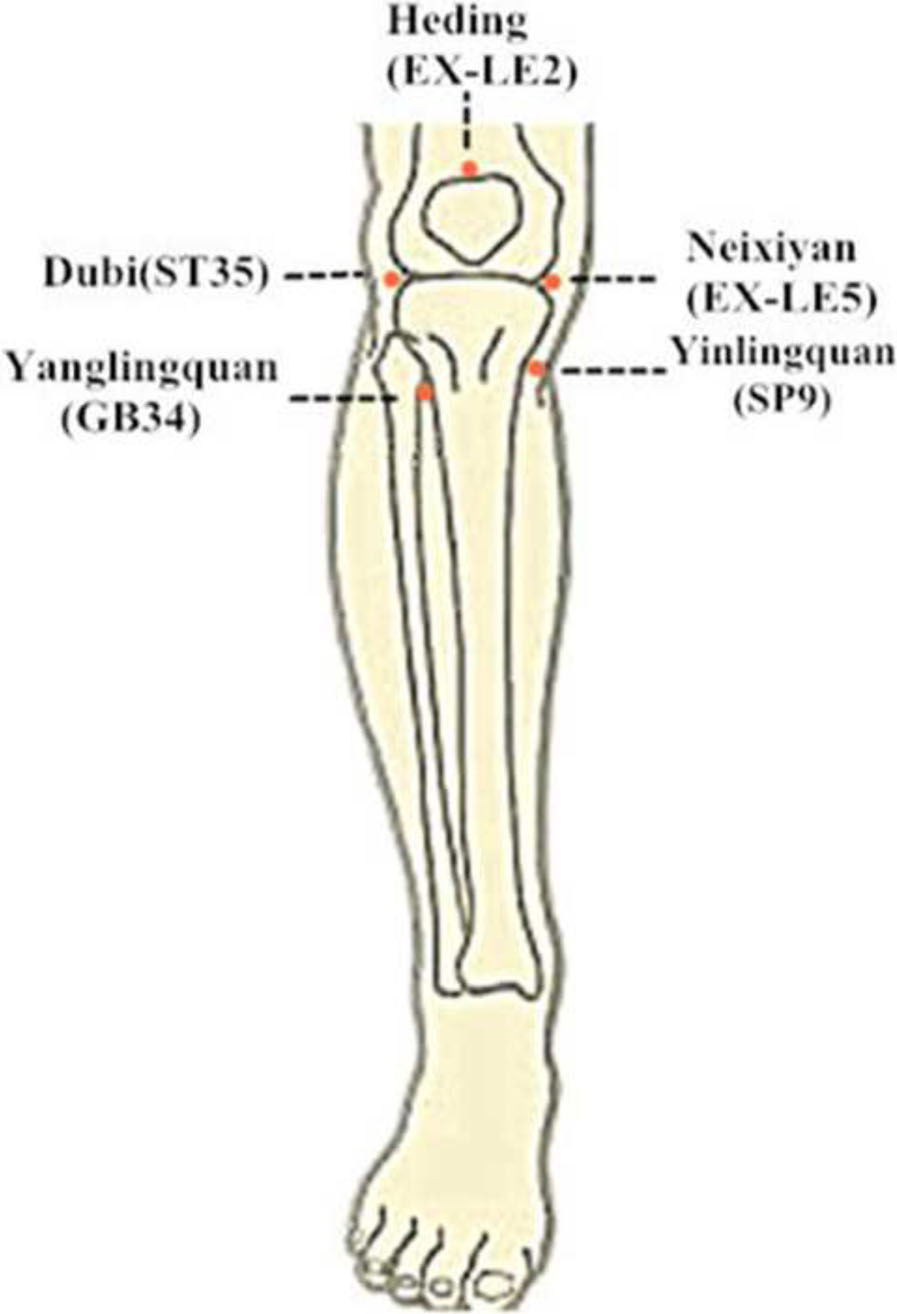
Location of acupoints. Five points are used for each patient. They are *Dubi* (ST35), *Neixiyan* (EX-LE5), *Heding* (EX-LE2), *Yinlingquan* (SP9), and *Yanglingquan* (GB 34)

The acupoints *Dubi* (ST35), *Neixiyan* (EX-LE5), *Heding* (EX-LE2), *Yinlingquan* (SP9), and *Yanglingquan* (GB 34) will be used for each patient. According to the National Standard of The People’s Republic of China. The Name and Location of Acupoints, issued in 2006 (GB/T 12346-2006), *Dubi* (ST35), also called *Waixiyan*, is located in a depression lateral to the patellar ligament. *Neixiyan* (EX-LE5) is located in the center of the depression of the patellar ligament of the knee and is opposite ST35. *Heding* (EX-LE2) is located in the depression above the midpoint of the superior patellar border. *Yinlingquan* (SP9) is located in the depression between the lower border of the medial condyle of the tibia and the medial border of the tibia. *Yanglingquan* (GB 34) is located in the depression anterior and inferior to the head of the fibula (Fig. 2) Additional file 2.

In the *De Qi* group, disposable acupuncture needles (Φ0.25 × 40 mm; Suzhou Dongbang Acupuncture Inc., Suzhou, China, 215144) will be inserted vertically to a depth of 25–30 mm after sterilization of the target acupoints. Then the needles will be manipulated using techniques including lifting, thrusting, twisting, and rotating, until *De Qi* is achieved. During the treatment session, the acupuncturist will manipulate the needles every 10 minutes to maintain the intensity of the *De Qi* sensations.

In the control group, shorter disposable acupuncture needles (Φ0.25 × 25 mm; Suzhou Dongbang Acupuncture Inc., Suzhou, China, 215144) will be inserted vertically to a depth of 1–2 mm and left in position for 30 minutes without any manipulation.

### Outcomes

#### Initial visit

The researchers will collect the clinical information of the patients during their initial visits, including age, sex, course of disease, and body mass index. The participants will not yet be assigned to a treatment group at this time Additional file 2.

#### Knee pain intensity

**Fig. 3:**
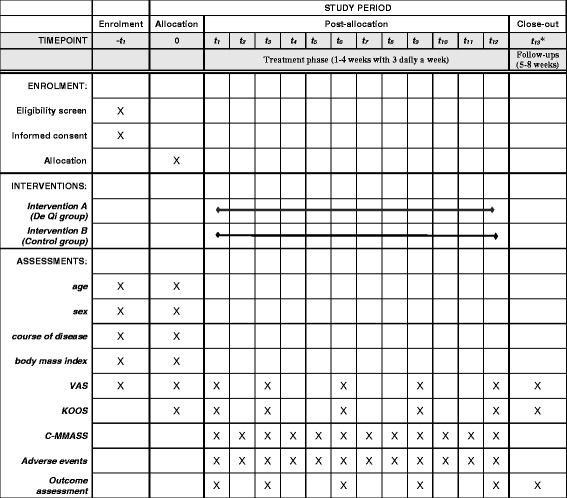
Planned visit schedule of enrollment, interventions, and assessments. Notes: *t_***13***_ is at the end of the follow-up (i.e., the 8th week). *VAS* visual analog scale, *KOOS* Knee injury and Osteoarthritis Outcome Score, *C-MMASS* Modified-Chinese version of the Massachusetts General Hospital Acupuncture Sensation Scale

To evaluate the efficacy of acupuncture treatment for KOA, knee pain intensity is measured via a 0–100 mm visual analog scale (VAS). The patient moves the slider on a 100-mm VAS ruler to indicate the intensity of knee pain that he/she is suffering, where the left end of the ruler marks “no pain,” and the right end marks “intolerable pain.” The distance (in millimeters) from the slider to the left end of the ruler is read directly from the back of the ruler by the researcher and is used as the VAS score for pain intensity, which is the primary outcome index in this study. The VAS score for knee pain intensity will be measured before each treatment and at the end of the 1^st^, 3^rd^, 6^th^, 9^th^, and 12^th^ treatments, as well as at the end of the follow-up visit (Fig. 3). Additional file 2

#### Knee injury and Osteoarthritis Outcome Score (KOOS)

The KOOS [23] is used as the secondary clinical outcome. The KOOS comprises of five subscales, each of which produces an outcome score. These subscales include pain, other relevant symptoms, activities of daily living (ADL), function in sports and recreation, and knee-related quality of life (QOL). Trained research assistants, who are blinded to the treatment, will measure the KOOS for all patients both at the baseline and at the end of the 1^st^, 3^rd^, 6^th^, 9^th^, and 12^th^ treatments and at the end of the follow-up (Fig. 3) Additional file 2. For each subscale, the outcome score will be normalized, where 0 indicates no symptoms and 10 indicates the most extreme symptoms [23].

#### Assessment of De Qi sensation induced by acupuncture

In our study, the assessment of *De Qi* sensation perceived by the patients is based on the Chinese version of the ”modified” Massachusetts General Hospital Acupuncture Sensation Scale (C-MMASS) (see Additional file 1). Before the trial, the concept of “*De Qi* and its correlated sensations” will be explained to all participants by the recorder. At the end of each treatment session after the removal of the needles, patients will be asked to fill in the C-MMASS form to evaluate the intensities of 11 acupuncture sensations: soreness, aching, deep pressure, heaviness, fullness/distension, tingling, numbness, dull pain, warmth, cold, and throbbing. The intensities of these sensations are evaluated on a 0–10 cm VAS, where 0 represents “none” and 10 represents “intolerably strong sense.” There is also an additional blank row at the end of the form that the patient can use to assess any other sensations. The MASS index will then be calculated.

#### Side effects

Any adverse events such as fainting, hematoma, and local infection during acupuncture will be recorded and analyzed Additional file 2.

### Statistics

All data will be analyzed using SPSS for Windows 17.0 Software (SPSS Inc., Chicago, IL, USA). For the main analysis, we will perform both an intent-to-treat principle and a per-protocol analysis. Measurement data will be described with mean ± standard deviation ($\overline{x}$ ± SD), and count data will be expressed with number of cases and percentages. All statistical analyses will use two-tailed tests, and the level of significance will be set at *p* < 0.05 Additional file 2. 

Demographic and baseline characteristics of study participants by randomization group will be analyzed by an independent sample *t* test, *t*' test, or nonparametric test. In the comparison between the *De Qi* and control groups, the primary outcome (VAS scores) and secondary outcome (KOOS, in the form of symptom scores) will adopt the method of within-subject 6 × 2 factorial repeated measures analysis of variance (ANOVA) to analyze the integral analgesic effect, where the factors are time (six levels: baseline, and at the end of the 1^st^, 3^rd^, 6^th^, 9^th^, and 12^th^ treatments) and group (two levels: *De Qi* group and the control group). The immediate analgesic effect will be evaluated by the VAS score after the first treatment; the accumulated analgesic effect will be rated by the VAS score after the last treatment. They will be analyzed by the independent sample *t* test and *t*' test or nonparametric test. In addition, the overall *De Qi* scores of each group, i.e., the sum of the scores of all sensations in each treatment, will be considered and analyzed with the primary outcome by a bivariate correlation test (described by *r* and *p*).

## Discussion

According to the theory of traditional Chinese medicine, *De Qi*, or the arrival of *Qi*, is pivotal for effective acupuncture therapy, which may alleviate the pain by eliminating the obstruction to the flow of *Qi*. Similar to pain, *De Qi* is also a subjective sensation, and it is affected by complicated factors such as emotion and cognition, which makes the study of *De Qi* and the therapeutic efficacy of acupuncture a great challenge. Recently, several groups of researchers have met this challenge with an evidence-based approach. In investigating the relationship between *De Qi* and the analgesic effect of acupuncture, Xiong et al. [24] confirmed the contribution of *De Qi* to the therapeutic outcome of acupuncture treatment in patients with primary dysmenorrhea. Their results showed a better relief of pain in the *De Qi* group than in the non-*De Qi* group, which led them to conclude that *De Qi* had a decisive role in the therapeutic effects of acupuncture. Xu et al. [25] found that a better therapy in patients with Bell’s palsy was achieved by the strengthened acupuncture stimulation to evoke *De Qi*, and the stronger the *De Qi* sensation, the better the therapeutic outcome. In addition, with healthy volunteers as the subjects, researchers also observed changes in the threshold of pressure- or heat-induced pain that was affected by *De Qi* sensation [26, 27], suggesting a relationship between the intensity of needling sensation and the pain threshold.

From the point of view of neurology, acupuncture, as an external stimulus, may affect the central nervous system through perception, thereby exerting its peripheral effects. At the end of the last century, researchers adopted the functional magnetic resonance imaging (fMRI) technology to explore the cerebral effects of acupuncture in multiple diseases such as stroke, Parkinson’s disease, and Alzheimer’s disease [28, 29]. Several groups attempted to unveil the difference in cerebral responses between *De Qi* and non-*De Qi* stimulations For example, Hui et al. [30] used this technology to investigate the influence of *De Qi* on activated brain regions. In their study, an inhibited state (i.e., negative activation) of blood oxygen level-dependent (BOLD) signal in the cerebral limbic system and paralimbic system was observed when the subjects perceived *De Qi* sensations such as soreness, numbness, and swelling. At this moment, the subject might feel relaxed and comfortable. The BOLD signal was reversed when the subjects perceived a sense of piercing pain instead of *De Qi* sensations. This suggested that *De Qi* sensations could to be visualized as cerebral signals through functional imaging. Furthermore, Asghar et al. [31] reported a synergistic signal attenuation in the middle temporal gyrus, the fusiform gyrus, and the lingual gyrus when the subjects sensed *De Qi* sensations. These regions are all related to the limbic-paralimbic-neocortical network (LPNN) [32]. Bai et al. [33] and Wu et al. [34] obtained similar results. Taken together, these findings suggested that a broad negative activation of the limbic system may be induced by the *De Qi* sensation during acupuncture. It has also been reported [35] that the limbic system is related to the release of multiple neurotransmitters, such as serotonin (5-HT) and dopamine, which may induce an analgesic effect during acupuncture and may underlie the neurophysiological mechanism of the role of *De Qi* in acupuncture.

The aim of this study is to explore the analgesic effect (including immediate and accumulated analgesic effects) of *De Qi* induced by acupuncture in KOA patients. Different needling depths with or without needle manipulation will be used to induce or avoid *De Qi*. The actual *De Qi* sensations will be assessed with a *De Qi* scale (C-MMASS). The correlation between the overall *De Qi* scores and VAS of knee pain will also be analyzed to reveal the contribution of *De Qi* to the therapeutic effects. This trial will help to provide a clinical basis for further investigation of the relationship between *De Qi* and the therapeutic efficacy of acupuncture.

### Trial status

This trial is currently in the process of treatment and follow-up, and it is expected to finish by the end of August. The work of the recruiting (88 participants) was completed on 20 May 2017.

## Additional files

Additional file 1: C-MMASS. (DOC 139 kb)

Additional file 2: SPIRIT checklist. (DOC 123 kb)

## Abbreviations

BOLD: Blood oxygen level-dependent
CI: Confidence interval
C-MMASS: Chinese version of the “modified” Massachusetts General Hospital Acupuncture Sensation Scale
fMRI: Functional Magnetic Resonance Imaging
ITT: Intention-to-treat
KOA: Knee osteoarthritis
LPNN: Limbic-paralimbic-neocortical network
PP: per-protocol
VAS: Visual analog scale
